# Comparison of surgically induced astigmatic changes after cataract surgery in post-Laser *in situ* keratomileusis corneas versus virgin eyes

**DOI:** 10.5935/0004-2749.20230070

**Published:** 2023

**Authors:** Melisa Zisan Karslioglu, Cem Kesim, Ayse Yildiz Tas, Murat Hasanreisoglu, Orkun Muftuoglu, Afsun Sahin

**Affiliations:** 1 Department of Ophthalmology, Koc University Hospital, Istanbul, Turkey; 2 Department of Ophthalmology, School of Medicine, Koc University, Istanbul, Turkey

**Keywords:** Cataract surgery, Keratomileusis, laser *in situ*, Refractive surgery, Surgically induced astigmatism, Vector analysis, Cirurgia de catarata, Ceratomileuse, excimer laser *in situ*, Cirurgia refrativa, Astigmatismo induzido cirurgicamente, Análise vetorial

## Abstract

**Purpose:**

Postoperative refraction in modern mi­croincision cataract surgery gained extra importance in patients with the previous laser-assisted *in situ* keratomileusis (LASIK) surgery. The surgically induced astigmatic changes in those eyes may differ not only in magnitude but also in direction compared to virgin corneas. This study aimed to compare the surgically induced astigmatic changes after microscopic cataract surgery between post-LASIK corneas and virgin eyes.

**Methods:**

Cases that underwent microincision cataract surgery in eyes with and without previous LASIK surgery were reviewed. The demographics, the axial length at cataract surgery, the central corneal thickness, spheric and cylindric values, keratometry readings, and postoperative posterior corneal astigmatism were retrospectively evaluated. A modified Alpins method was used for astigmatic vector analysis, and baseline astigmatism, surgically induced astigmatism, difference vector, flattening effect, and torque were assessed.

**Results:**

A total of 42 eyes from 24 subjects was evaluated. Group I consisted of 14 eyes with the previous LASIK, and Group II included 28 eyes without any refractive surgery. Preoperative mean central corneal thickness in Group I was significantly thinner (p=0.012). There was no significant difference in baseline astigmatism between the groups regarding magnitude and power vectors. After microincision cataract surgery, there were no significant differences in mean spheric and cylindric values and mean keratometry readings (all p>0.05). However, surgically induced astigmatism and difference vector were significantly higher on J_45_ vector component in post-LASIK eyes and microincision cataract surgery steepening effect on post-LASIK corneas was significantly higher than those in virgin eyes (p=0.001, p=0.002 and p=0.018, respectively).

**Conclusions:**

Cataract surgery has steepened the corneas in both groups with a significantly higher steepening effect in post-LASIK eyes. Certainly, corneal topography cataract surgery is particularly helpful to provide more precise surgically induced astigmatism interpretations.

## Abstract (pt)

**Objetivo:**

A refração pós-operatória na cirurgia mo­derna de catarata por microincisão ganha ainda mais importância em pacientes com cirurgia prévia de ceratomileuse *in situ* assistida por laser (LASIK). As alterações astigmáticas induzidas cirurgicamente nesses olhos podem diferir não apenas em magnitude, mas também em direção em comparação com córneas virgens. O objetivo deste estudo foi comparar as alterações astigmáticas induzidas cirurgicamente após cirurgia de catarata por microincisão entre córneas pós-LASIK e olhos virgens.

**Métodos:**

Foi revisada uma série de casos de cirurgia de catarata por microincisão em olhos com e sem cirurgia LASIK anterior. Os dados demográficos, o comprimento axial no momento da cirurgia de catarata, a espessura central da córnea, os valores esféricos e cilíndricos, as leituras da ceratometria e o astigmatismo corneano posterior pós-operatório foram avaliados retrospectivamente. O método Alpins modificado foi usado para análise vetorial astigmática e foram avaliados o astigmatismo basal, o astigmatismo induzido cirurgicamente, o vetor de diferença, o efeito de achatamento e o torque.

**Resultados:**

Ao todo, 42 olhos de 24 indivíduos foram avaliados. O Grupo I consistiu em 14 olhos com LASIK prévio; o Grupo II incluiu 28 olhos sem qualquer cirurgia refrativa. A média da espessura corneana central pré-operatória no Grupo I foi significativamente mais fina (p=0,012). Não houve diferença significativa no astigmatismo basal entre os grupos em termos de magnitude e vetores de potência. Após a cirurgia de catarata por microincisão, não houve diferenças significativas nos valores médios esféricos, cilíndricos e leituras médias de ceratometria (todos com p>0,05). No entanto, o astigmatismo induzido cirurgicamente e o vetor de diferença foram significativamente maiores no componente do vetor J_45_ em olhos pós-LASIK, e o efeito de aumento da inclinação pela cirurgia de catarata por microincisão nas córneas pós-LASIK foi significativo em comparação com olhos virgens (p=0,001, p=0,002 e p=0,018, respectivamente). **Conclusões:** A cirurgia de catarata aumentou a inclinação das córneas em ambos os grupos, sendo esse aumento significativamente maior nos olhos pós-LASIK. Certamente, a topografia da córnea antes da cirurgia de catarata é particularmente útil para fornecer interpretações mais precisas do astigmatismo induzido cirurgicamente.

## INTRODUCTION

Refractive surgery has been performed on millions of people over the years. These patients underwent laser-assisted *in situ* keratomileusis (LASIK) or photorefractive keratectomy (PRK) to become as glasses-independent as possible. More and more such patients are now undergoing cataract surgery and expect the same visual freedom they had before the disease and surgery. One of the important parameters to ensure patient satisfaction is residual postoperative astigmatism. With premium intraocular lenses development, astigmatism management during cataract surgery gained more importance to meet patient expectations. It is well-known that residual astigmatism greater than 0.50 diopters after cataract surgery can reduce uncorrected visual acuity^(1)^. Therefore, ophthalmologists should know surgically induced corneal changes after microincision cataract surgery. This becomes more important in patients who have undergone LASIK surgery.

Since LASIK was reported by Pallaris et al. in 1990^(2)^, the patients who had undergone LASIK surgery have aged over the past 30 years^(3)^, and cataract surgery became necessary. Unexpected refractive outcomes after cataract surgery in this population have presented a new challenge in postoperative definite refractive assessment because of their non-virgin corneas^(4)^. A patient with high myopia who has also had LASIK still has high myopia anatomy, although he or she no longer has the refractive high myopia error. This introduces several complicating factors, including axial length measurement errors, intraocular lens power calculation formula errors, intraoperative reverse pupillary block, and greater corneal and scleral elasticity. As the eye wall tends to be more elastic, extreme care must be taken when making a clear corneal incision.

Corneal biomechanics after refractive surgeries may be unpredictable and has not been fully understood. Therefore, we hypothesize that corneal astigmatism changes can differ from the changes in virgin eyes after cataract surgery.

This study aimed to investigate whether the previous refractive surgery has a different effect on refractive rehabilitation in eyes after cataract surgery. By using modified astigmatic vector analysis, we analyzed the surgically induced astigmatic changes not only in magnitude but also in direction to compare these parameters in post-LASIK corneas versus virgin eyes after cataract surgery, contributing to the current literature.

## METHODS

### Patient population

This retrospective study was approved by the institutional review board of Koc University Committee on Human Research (No 2020. 295.IRB1.103) and conforms with the principles and applicable guidelines for protecting human subjects in biomedical research. Between September 2019 and December 2020, we reviewed consecutive cases of microincision cataract surgery in the eyes that had undergone LASIK surgery before. The demographics, including age and sex, the axial length at cataract surgery, the central corneal thickness, spheric and cylindric values, and keratometry readings were retrospectively evaluated. We also calculated the posto­perative posterior corneal astigmatism by subtracting corneal astigmatism from the total actual astigmatism, assuming that the lens-induced astigmatism was zero after cataract surgery. An autorefractometer and a partial coherence laser interferometry were used in recording axial length, central corneal thickness, keratometry readings, and corneal astigmatism.

### Surgical technique in phacoemulsification

All surgeries were performed by a single right-handed surgeon under topical proparacaine hydrochloride anesthesia. After phacoemulsification, acrylic hydrophilic foldable intraocular lenses were implanted. Following his habits, the surgeon sit at the 9 o’clock position for the right eyes and at the 3 o’clock for the left eyes. The two-side ports were made with 20-gage angled side-port blades at 90º to the planned main clear corneal incision in both eyes at 12 and 6 o’clock. A main clear corneal three-planar horizontal incision of about 1 mm inside the limbus was made with a 2.2-mm diamond knife. The divide and conquer approach technique was used for nuclear fragmentation, and the rest of the surgical steps were performed as in routine phacoemulsification. The patients were recommended the same topical antibiotic eye drops (moxifloxacin 0.5% ophthalmic solution 5 times daily for 7 days) and topical steroid eye drops (dexamethasone 0.1% 5 times daily for a month) for the postoperative treatment.

### Astigmatic vectorial analysis

We used a modified Alpins method for astigmatic vector analysis and defined the vectorial parameters used in the calculation for analysis as follows.

**(i) the baseline astigmatism (BA):** the astigmatic value that was present before surgery,

**(ii) the surgically induced astigmatism (SIA):** the astigmatic change that was induced in reality,

**(iii) the difference vector (DV):** remaining astigmatism and the summary of the astigmatic error considering both magnitude and axis,

**(iv) the flattening effect (FE):** the force that lies on the intended axis without rotating effect, which might have flattening (­) or steepening (+) effect,

**(v) the torque (TRQ):** the force that lies at 45 degrees to that intended axis which is ineffective in reducing pre-existing corneal astigmatism and might be clockwise or counterclockwise. The magnitudes of meridional and torsional components of each astigmatic vector are given as J_0_ and J_45_ power vectors as proposed by Thibos et al.^(5)^ J_0_ is local astigmatism at 0 and 90 degrees, and J_45_ is local astigmatism at 45 degrees and 135 degrees.

Even though most of the patients could not remember the exact date of their previous LASIK surgery, the time they reported was more than 25 years. Therefore, we could not reach the keratometric data before the refractive surgery. Our study assumed the current data before cataract surgery as the baseline for post-LASIK eyes. The BAs, the SIA, the DV, the FE, and the torque^(6)^ of the astigmatism were assessed in both post-LASIK corneas and virgin eyes after microincision cataract surgery. A negative value for the FE indicated that the vector of SIA flattened the BAs. In contrast, a positive value showed that the vector of SIA steepened the BAs.

### Statistical analysis

All statistical analysis was performed using Statistical Package for the Social Sciences statistical software version 22. One-way analysis of variance and Pearson’s chi-squared test were used for categorical variants. Other values were reported as mean ± standard deviation, and Mann-Whitney U test and Kruskal-Wallis test were used for continuous variables. All p values less than 0.05 were considered statistically significant (p<0.05).

## RESULTS

A total of 42 eyes from 24 subjects was included in the study. While Group I consisted of 14 post-LASIK eyes, Group II included 28 virgin eyes who underwent cataract surgery. The mean age at cataract surgery in Group I and II was 67.9 ± 8.6 years and 64.4 ± 7.6 years, respectively (p=0.120). There was no significant difference between the groups regarding sex (female: male ratio in Group I, 4:3; in Group II, 7:10, p=0.125).

The preoperative and postoperative data of the eyes in the two groups are shown in table 1 and table 2, respectively. There were no significant differences in preoperative mean axial length, spheric and cylindric values, and mean keratometry readings (all p>0.05, Table 1), and as expected, the preoperative mean central corneal thickness in Group I was significantly thinner than those in Group II (527 ± 25 µm and 555 ± 40 µm, respectively, p=0.012, Table 1).

**Table 1:** The preoperative data of the eyes in the study groups

	Group 1	Group 2	
Preoperative Data	Post-LASIK eyes (n=14) (Mean ± SD)	Virgin eyes (n=28) (Mean ± SD)	p-value
AL (mm)	23.85 ± 1.15	23.93 ± 1.64	0.730
CCT (µm)	527 ± 25	555 ± 40	0.012*
Sphere (D)	1.46 ± 1.51	-0.36 ± 3.84	0.204
Cylinder (D)	-0.69 ± 0.34	-1.00 ± 0.61	0.099
SE	1.15 ± 1.43	3.90 ± 2.99	0.216
K readings (D)			
• K1	42.68 ± 1.97	43.18 ± 1.53	0.539
• K2	43.36 ± 2.16	43.52 ± 1.49	0.831

LASIK= laser *in situ* keratomileusis; AL, axial length; CCT= central corneal thickness; D, diopter; SE= spheric equivalent; K= keratometry; SD= standard deviation;

mm= millimeter; µm= micrometer.

Differences shown with an asterisk (*) were statistically significant, with p-value <0.05.

**Table 2:** The postoperative data of the eyes in the study groups

	Group 1	Group 2	
Postoperative data	Post-LASIK eyes (n=14) (Mean ± SD)	Virgin eyes (n=28) (Mean ± SD)	p-value
Sphere (D)	-0.07 ± 0.87	-0.01 ± 0.45	0.705
Cylinder (D)	-0.73 ± 0.56	-0.65 ± 0.49	0.400
SE	-0.43 ± 0.98	-0.35 ± 0.72	0.619
K readings (D)			
• K1	41.72 ± 2.27	42.46 ± 1.88	0.138
• K2	42.97 ± 2.09	43.45 ± 1.80	0.297
Posterior Corneal Astigmatism	0.81 ± 0.58	0.73 ± 0.50	0.769

LASIK= laser *in situ keratomileusis*; D= diopter; SE= spheric equivalent; K= keratometry SD= standard deviation.

Differences shown with an asterisk (*) were statistically significant, with p-value <0.05.

After microincision cataract surgery, there were no significant differences in postoperative mean spheric and cylindric values and mean keratometry readings (all p>0.05, Table 2). The postoperative posterior corneal astigmatism was higher in post-LASIK eyes; however, the difference was not statistically significant between the groups (p=0.769, Table 2).

The vector analysis results between the two study groups, including the mean BAs, SIA, DV, FE, and torque are shown in table 3. There was no significant difference in BAs between the groups regarding magnitude and power vectors. However, the SIA and DV were significantly higher on the J_45_ power vector component in post-LASIK eyes than those in virgin eyes (p=0.001 and p=0.002, respectively, Table 3). The positive values of the FE showed that the cataract surgery had steepened the BAs in both groups, with a significantly more steepening effect in post-LASIK eyes (p=0.018, Table 3).

**Table 3:** Astigmatism vector analysis between the study groups

	Group 1	Group 2	
Parameters	Post-LASIK eyes (n=14) (Mean ± SD)	Virgin eyes (n=28) (Mean ± SD)	p-value
BA magnitude	0.68	0.71	0.947
J_0_	0.15	0.26	0.122
J_45_	0.27	0.19	0.420
SIA magnitude	0.78	0.43	0.052
J_0_	0.31	0.18	0.076
J_45_	0.22	0.08	0.001*
DV magnitude	1.26	0.86	0.113
J_0_	0.37	0.35	0.742
J_45_	0.46	0.19	0.002*
FE (­)/ SE (+)	0.66 ± 0.64	0.23 ± 0.41	0.018*
TRQ	0.25 ± 0.19	0.21 ± 0.17	0.589

LASIK= laser *in situ keratomileusis*; BA, baseline astigmatism; SIA= surgically induced astigmatism; DV= difference vector; FE= flattening effect; SE= steepening effect; TRQ= torque; J_0_= Jackson cross-cylinder, astigmatism axes at 0 degrees and 90 degrees; J_45_= Jackson cross-cylinder, astigmatism axes at 45 degrees and 135 degrees.

Differences shown with an asterisk (*) were statistically significant, with p-value <0.05.

## DISCUSSION

Generally, all cataract and refractive surgeries aim not only to treat patients but also to achieve better well-being. However, a satisfying visual rehabilitation may be difficult to achieve in these surgeries where too many variable factors are involved. In this study, we found that post-LASIK patients who underwent cataract surgery had higher SIA than the control group. Also, we revealed that having cataract surgery provided a higher steepening effect in post-LASIK corneas compared to virgin eyes.

SIA is considered one of the challenging factors in postoperative visual rehabilitation. It is defined as the difference between preoperative and postoperative astigmatism and can also be used in manifest refraction and corneal analysis^(7)^. In other words, it is an astigmatism variant caused by the healing and scar formation occurring at the incision site^(6)^. Many different factors playing a role in SIA formation have been investigated in the literature. Theodoulidou et al. evaluated the surgeon factor in cataract surgery with phacoemulsification and compared SIA between four different experienced surgeons performing the same main 3-step clear corneal incision in 3-mm width and a side-port incision in 1-mm width^(8)^. SIA was calculated by vector analysis via the Alpins method^(9)^. The authors found no significant difference in SIA among surgeons. Thus, they declared that SIA might be more related to preoperative keratometric values and incision characteristics instead of surgeon factor. In our study, the same surgeon performed all surgeries; thus, the surgeon factor was excluded. However, although the comparison of preoperative keratometric readings and corneal incisions was statistically insignificant between the groups, the significant difference in SIA found among our study participants directed us to focus on prior LASIK surgery.

Differently, Ferreira et al. compared SIA, the FE of wound healing, and the torque of astigmatism caused by femtosecond laser and manual clear corneal incisions^(6)^. Alpins method was used for astigmatic vector analysis, similar to our study^(9)^. Although femtosecond laser clear corneal incisions showed more reproducible standardized architecture, more precise location, less wound deformation, less endothelial damage, and smaller SIA, no significant difference was identified between femtosecond laser and manual clear corneal incisions in their study. The main outcomes evaluated in our study were nearly similar to those in Ferreira’s study. We concluded that significant differences in SIA and FE in our results were secondary to structural corneal changes due to previous LASIK surgery because cataract surgeries were performed by the same surgeon with the same incision preference and habit.

Previously, in a randomized controlled clinical trial, the torque and FE of temporal and on-axis clear corneal incisions in phacoemulsification were compared by Borasio et al.^(10)^ using the Alpins method. In the eyes with preoperative astigmatism below 2.60 diopters, the temporal clear corneal incisions were associated with lesser torque and induced less FE in postoperative astigmatism compared to on-axis clear corneal incisions. In our study, all eyes had undergone temporal clear corneal incisions. Also, no significant difference was found regarding torque between the study groups; however, a greater steepening effect was calculated in the post-LASIK eyes.

Our study also evaluated posterior corneal astigmatism, which is astigmatism that compensates for 31%^(11)^ of anterior corneal surface astigmatism. In a retrospective series including 132 eyes, Park et al.^(12)^ evaluated not only the torque and FE of steep meridian incisions but also the effect of preoperative posterior corneal astigmatism on postoperative total corneal astigmatism after cataract surgery. The authors found that in eyes with greater posterior corneal with-the-rule astigmatism and greater superior corneal thickness, steep meridian incisions may cause a significant torsional effect and make the posterior cornea more against-the-rule astigmatic via shifting the steeper axis. Differently, in our study, temporal incisions were performed in all eyes, and although the difference was insignificant, postoperative posterior corneal astigmatism was greater in post-LASIK corneas. SIA was also higher in magnitude, and a significant shift toward the oblique axes was observed in post-LASIK eyes compared to virgin eyes after cataract surgery. We thought that these findings might be caused by corneal integrity disruption induced by previous corneal refractive surgery.

However, Zhang et al.^(13)^ investigated the displacement in the posterior corneal surface shape after LASIK and, on the contrary, reported that this was a time-dependent process; in other words, the posterior corneal surface returned to untouched state 6 months after the surgery. Bao et al.^(14)^ also investigated regional corneal changes after femtosecond laser-assisted LASIK and stated that the changes differed in different regions (central, pericentral, and peripheral). Similar to the study of Zhang et al.^(13)^, during the 6-month follow-up, they reported that these changes also tended to reverse, but they highlighted that posterior corneal astigmatism remained stable. Supporting those findings, in our study, although the postoperative posterior corneal astigmatism was higher in post-LASIK eyes, no significant difference was found between the study groups. This might be because our patients had undergone refractive surgery approximately 25 years ago and both mean keratometric readings and autorefractive values were statistically insignificant before the cataract surgery.

In the light of previous studies, it has been proven that the corneal biomechanical strength deteriorates after refractive surgery and corneal hysteresis decreases^(15)^. Denoyer et al. stated that the elasticity modulates the optical quality together with wound healing corneal response after cataract surgery^(16)^. The incision architecture, optical analysis of astigmatism with high-order aberrations, and biomechanical changes were assessed by spectral-domain anterior segment optical coherence tomography, Scheimpflug topography, and ocular response analyzer, respectively, in their study. The authors identified lower final SIA in patients with higher preoperative corneal hysteresis despite a large incision. In comparison, higher final SIA was found in patients with lower preoperative corneal hysteresis despite a microincision. Since corneal hysteresis has proven to decrease following LASIK^(17)^ and SIA has been negatively correlated with corneal hysteresis, in accordance with the literature, a significant increase in the SIA was calculated in post-LASIK corneas compared to virgin eyes in our study.

Our study has a few limitations. First, a relatively small sample was studied. This was because there were relatively few cataractous post-LASIK eyes in the population. The second limitation was the absence of preoperative corneal topography imaging in some participants because our study is retrospective, and corneal topography imaging before cataract surgery was not routinely applied in our clinical practice unless toric intraocular lens implantation was planned. Another drawback was that the patients were further examined to understand corneal biomechanics regarding corneal hysteresis and corneal resistance factor.

Nevertheless, this study showed that SIA calculated after microincision cataract surgery was higher in post-LASIK corneas and cataract surgery had a higher steepening effect on post-LASIK corneas compared to virgin eyes.
